# Spicing Up Vegetables: Consumer Attitudes and the Impact of Seasoning on Vegetable Consumption in a Cafeteria Setting

**DOI:** 10.1093/nutrit/nuaf246

**Published:** 2026-05-26

**Authors:** Carter W Phillips, Guy H Johnson

**Affiliations:** Department of Food Science and Human Nutrition, University of Illinois at Urbana-Champaign, Champaign, IL 61820, United States; Department of Food Science and Human Nutrition, University of Illinois at Urbana-Champaign, Champaign, IL 61820, United States

**Keywords:** vegetables, behavior, diet, aging, inflammation

## Abstract

Vegetables are a key component of a healthy diet; however, their consumption in the United States has remained well below recommended amounts for at least 20 years. The consumption of vegetables is determined by a complex array of factors, including knowledge, beliefs, affordability, and cultural considerations. A potentially important barrier to increased consumption is the perception that vegetables do not deliver on taste. Spices and herbs are potential solutions to such a barrier, because they add flavor without contributing meaningful amounts of calories or nutrients requiring limitation (eg, sodium, saturated fat, added sugars). Spices and herbs may also be a factor in cultural and other aspects that affect the acceptance of vegetables. Therefore, a series of experiments were conducted to determine whether the use of such seasonings would increase the acceptability and/or consumption of vegetables in a cafeteria setting. Qualitative data from focus groups were collected to learn why and how consumers utilize spices and herbs as well as vegetables. Quantitative data from an on-line survey of U.S. consumers as well as controlled sensory panels were also collected to assess current spice/herb preferences, frequency of use, and beliefs about their potential use when cooking vegetables. These studies were followed by 3 cafeteria-based experiments to examine the purchase, liking, and consumption of seasoned vs unseasoned vegetables. It was found that consumers were more likely to select seasoned vegetables when offered as a free side dish, or when sold for $1. Liking was high for both seasoned and unseasoned vegetables, with few differences between the 2. Plate waste was low for both forms, with no significant differences between standard (1/2 cup, 113 g) or larger (1 cup, 227 g) serving sizes. Collectively, these data suggest that combining seasoning with larger serving sizes may be a viable strategy for increasing vegetable consumption in a commercial cafeteria setting.

## INTRODUCTION

This article appears as part of the supplement, “The Role of Spices and Herbs in Supporting Healthy Diets and Improving Nutritional Status,” sponsored by the McCormic Science Institute.

Fruits and vegetables are essential components of a healthy diet, as important sources of vitamins, dietary fiber, and other bioactive compounds. In addition to providing daily required nutrients, numerous studies have found that sufficient fruit and vegetable intake helps to reduce the risk of many diseases, such as cancer,^1^^,^^2^ stroke,^3^^,^^4^ and cardiovascular diseases.^5^ Nevertheless, most Americans do not meet the dietary recommendations for these foods. The 2020–2025 *Dietary Guidelines for Americans* (DGAs)^6^ recommend 2.5 cups of vegetables and 2 cups of fruit per day for a 2000-calorie diet. However, recent intakes have remained well below these recommendations. According to the National Health and Nutrition Examination Survey,^7^ the average intake of vegetables was 1.5 cups per day in 2010–2011. Unfortunately, intakes reported by the 2020–2025 DGAs remain essentially unchanged.

One of the major barriers to the consumption of vegetables and other healthy foods is the perception that they do not deliver on taste. Data from the International Food Information Council Diet and Health Survey^8^ show that “taste” has remained the most important factor that determines the foods consumers purchase and consume. Spices and herbs add flavor to foods without contributing meaningful amounts of sodium, saturated fat, added sugars or calories. Furthermore, seasoning vegetables with spices and herbs may increase their acceptance and consumption. For example, mean consumption at an inner-city high school cafeteria increased by 18.2% compared with baseline when spices and herbs were used to season them.^9^

The purpose of this research was to understand the potential of spices and herbs to affect the purchase, intake, liking and consumption of vegetables in a commercial cafeteria on the campus of the University of Illinois in Urbana. The approach was to first conduct focus groups among consumers to determine attitudes, self-efficacy, and behaviors related to liking and usage of vegetables and herbs and spices, as well as strategies to increase vegetable intake.^10^ Quantitative data were then collected using an on-line questionnaire to assess current spice and herb liking, use frequency, whether such ingredients were used in cooking vegetables, and the belief that spices and herbs could be used when cooking vegetables.^11^ Furthermore, controlled sensory experiments were conducted to determine the liking of seasoned vs unseasoned vegetables among 749 panelists.^12^ These data informed a series of experiments performed in a commercial cafeteria setting to see whether adding spices and herbs could affect consumer behavior.^13–15^

### Qualitative Data

Qualitative data on vegetable consumption and the potential use of spices and herbs to increase consumption were obtained from *n* = 54 subjects on campus.^10^ Adults, aged 18 years or older, were recruited through a university social media website. Recruits had to have eaten carrots, broccoli, cauliflower, peas, squash, zucchini, Brussel sprouts, corn, potatoes, okra, or tomatoes at least once in the previous 2 months to be considered for the study. There were no other exclusion criteria. Sex, age, and residence questions were asked during screening to stratify participants across focus groups as needed. Invitations to participate were sent by email, asking for availability. Four to 8 participants were assigned to each conference call focus group time. Participants were called simultaneously by the moderator. Participants selected fictitious names to preserve confidentiality. Focus groups lasted an average of 38 minutes, after which participants were compensated with a $70 gift card.

When asked to name their 3 favorite vegetables, 39 different vegetables were mentioned, with broccoli, peppers (green or red), and asparagus most often mentioned. Participants primarily cited flavor as the reason for picking their favorite vegetable (*n* = 28), followed by versatility of a vegetable’s use (*n* = 15). Most participants ate vegetables at home (*n* = 33), with family (*n* = 28) or as part of the evening meal (*n* = 41).

A number of themes emerged from the focus groups and were categorized into 2 main ideas: motivations for eating vegetables (attitudes) and strategies to increase vegetable consumption (self-efficacy and behavioral control).

Family was the predominant thematic reason for eating vegetables. Health was identified as the main reason for consuming vegetables, both their own health as well as the health of their parents and others. Children emerged as a motivator for serving vegetables at meals. Many stated their upbringing led them to believe that vegetables were a necessary part of the family’s evening meal. Parents wanted to establish nutritious foods as part of their children’s lives and form good dietary habits.

The primary strategy that was suggested to increase vegetable consumption was to present new ideas or items to consumers. Ideas included sampling or tasting opportunities, providing use and meal pairing suggestions, and presenting new preparation techniques. Participants noted that in-store tastings introduced them to now-favorite vegetables of which they were previously unaware or had felt intimidated about. Respondents reported that herbs and spices can add flavor to a vegetable dish, but no one mentioned using herbs and spices to increase vegetable consumption. However, many participants discussed eating vegetables in ethnic dishes, both at home and at restaurants, which typically use herbs and spices. Notably, Mexican (*n* = 15) and Asian (*n* = 14) dishes were popular choices.

In conclusion, the qualitative data from focus groups found that vegetable intake has a family focus that should be emphasized to increase intake. Efforts to increase intake could emphasize flavor and versatile preparation methods through tasting opportunities or educational demonstrations.

### Quantitative Data

Quantitative data were obtained using an online survey administered through Qualtrics, LLC.^11^ Qualtrics recruited participants from an existing panel, using nonprobabilistic (quota) sampling in an effort to match sample characteristics to the US population in terms of sex, age, income, and geographic region; quotas for additional variables such as education and race/ethnicity were not included due to budgetary constraints. The sample was approximately equally distributed for sex, geographic location, education, and age, with the exception of the “over 70 years and older” group. The majority of participants were white/Caucasian. For data analysis purposes, participants were divided into groups for age (18–29; 30–49; 50 years and older), income (less than $50 000; $50 000–$99 999; $100 000 or more), education level (some high school or high school diploma/General Educational Development; some college or associates/technical degree; Bachelor or graduate degree), and race (white/Caucasian; African–American or Hispanic; Asian/Pacific Islander, or Other).

**Figure 1.**
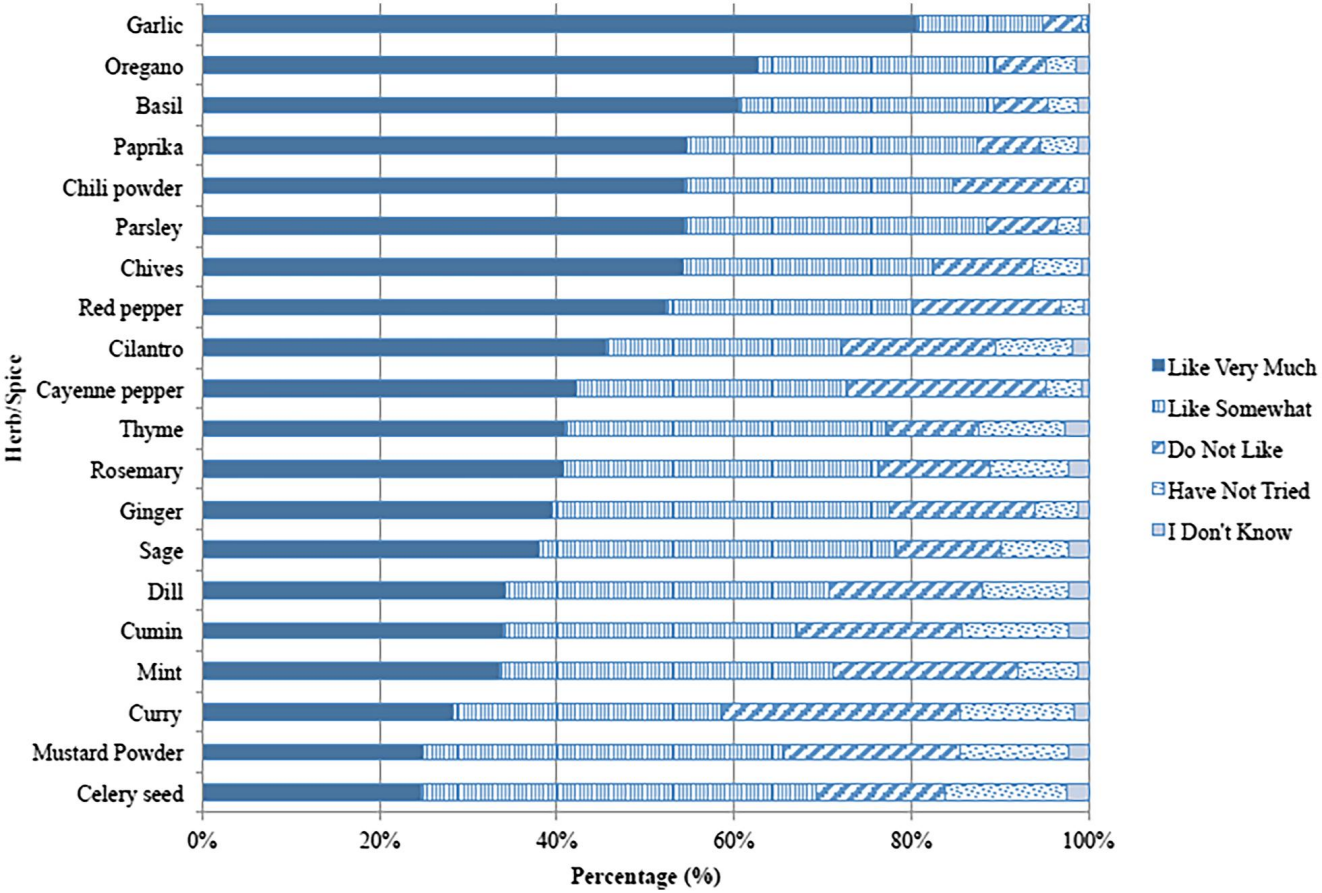
Survey Participants’ Liking of 20 Spices and Herbs (*N*=1042). Source: Nikolaus et al (2017)^11^

The overall liking of spices and herbs is displayed in Figure 1. The most commonly liked spices and herbs among all respondents were garlic, oregano, basil, and paprika. The liking of specific spices and herbs varied in accordance with numerous factors, including gender, income, educational background, geographic location, and ethnicity. This diversity of liking of spices and herbs suggests that their use to increase diet quality is complex, and we must accommodate such factors in order to achieve positive outcomes.

With respect to the use of spices and herbs while cooking vegetables, among high-frequency users, a higher proportion of women used garlic; men more often used cayenne pepper, curry, mint, mustard powder, red pepper, and (all *P *< .05). A greater proportion of younger respondents (18–29 years) used 19 of the 20 spices and herbs listed (the only exception was garlic) more often for cooking with vegetables than other age groups (all *P *< .001). Basil, chives, mint, oregano, sage, and thyme were used most by high-income users ($100 000 or more) compared with other income groups (all *P *< .05). Garlic was used more frequently by respondents with an income of $50 000–$99 999 (*P *< .001). Respondents with some college or an associate’s degree used mint more often than other education groups (*P *< .05). The northeast United States had a significantly larger proportion of high frequency users of parsley relative to other geographic regions (*P *< .01). There were significantly greater proportions of high-frequency spices and herbs users for 19 of the 20 spices and herbs among respondents who identified as Asian/Pacific Islander or Other (all *P* < .05). Cilantro, however, was more frequently used by African–American and Hispanic or Asian/Pacific Islander and Other races, compared with white/Caucasian respondents (*P *< .001).

The predictors of use of spices and herbs when cooking vegetables provide additional insights. Overall, 86.1% of respondents indicated they currently used spices and herbs when cooking vegetables at home. The logistic regression model predicting the use of spices and herbs when cooking vegetables at home included sex, age, education level, income, geographic region, and race as variables (Table 1). Respondents who did not cook vegetables at home (24 participants) were excluded from the analysis. The Cox and Snell R^2^ indicated that the model explained 3.4% of the variability in the use of spices and herbs while cooking vegetables. Female respondents were 1.6 times more likely than males to use spices and herbs when cooking vegetables (*P *< .05). No other predictors significantly predicted the use of spices and herbs when cooking vegetables. Despite the high use of spices and herbs for seasoning vegetables at home, as noted previously, the intake of such foods falls well below dietary recommendations. Additional data on the serving size and frequency of consumption of seasoned vegetables would be required to further explore this apparent inconsistency.

**Table 1.** Odds Ratios (ORS) and 95% CIS for Using Spices and Herbs When Cooking Vegetables at Home among Survey Participants (*N* = 1018)

Variable	OR [95% CI]
Sex: Female^a^	1.62* [1.11–2.36]
Age: 18–29 years^b^	1.64 [0.96–2.82]
Age: 30–49 years^b^	1.27 [0.85–1.90]
Income: Less than $50 000^c^	0.65 [0.37–1.16]
Income: $50 000–$99 999^c^	1.03 [0.57–1.87]
Education: Some High School or High School Diploma/General Educational Development^d^	0.61 [0.37–1/01]
Education: Some College or Associates/Technical Degree^d^	1.02 [0.63–1.64]
Region: Northeast^e^	0.83 [0.45–1.52]
Region: Midwest^e^	0.81 [0.46–1.42]
Region: South^e^	0.71 [0.43–1.18]
Race: White/Caucasian^f^	0.43 [0.16–1.10]
Race: African–American or Hispanic^f^	0.83 [0.29–2.42]

Source: Nikolaus et al (2017).^11^

* *P *< .05.

a Relative to male respondents.

b Relative to age: 50 years and older.

c Relative to income: $100 000 or more.

d Relative to education; Bachelor or Graduate/Professional Degree.

e Relative to region: West.

f Relative to race: Asian/Pacific Islander or Other.

In conclusion, these survey data suggest that low-income, male, older (≥50 years), and white/Caucasian respondents were identified as the target audiences that may benefit the most from interventions encouraging the use of spices and herbs with vegetables to increase consumption. The cafeteria-based experiments discussed below did not attempt to recruit such subjects, because these studies were conducted among all patrons using the cafeteria. Nevertheless, it is critical to account for the socio-demographic characteristics of the audience when designing such interventions, and customization according to such groups may be a key component of strategies to improve vegetable intake in future studies.

### Sensory Testing of Seasoned Vegetables

**Figure 2.**
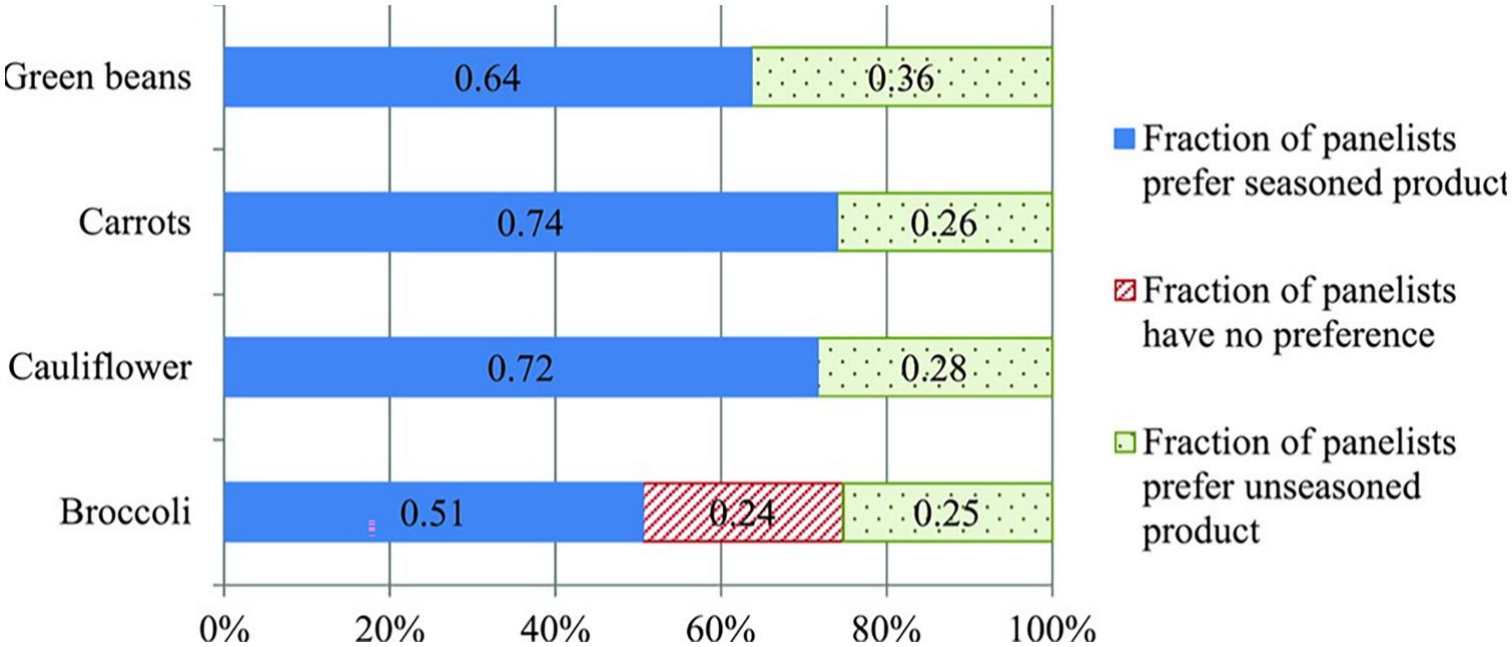
Distribution of Panelists on their Preference of Seasoned or Unseasoned Vegetables. Source: Feng et al (2018)^12^

Controlled experiments were conducted to determine the liking of seasoned vs unseasoned vegetables among 749 panelists.^12^ Adult panelists were screened and recruited as specific vegetable likers of the vegetable being tested or general vegetable likers. Four sessions were designed to evaluate the effect of seasoning within each type of vegetable, including broccoli, cauliflower, carrot, and green bean. Each panelist was only allowed to participate in one test session to evaluate only one vegetable type, so as to mitigate potential learning effect. Overall, the results showed that seasoned vegetables were significantly preferred over unseasoned vegetables (*P *< .001), indicating the sensory properties were significantly improved with seasoning, as shown in Figure 2.

It was concluded, “The findings from this study demonstrated that seasoned vegetables feature significantly greater acceptance rating compared to unseasoned vegetables. It was also found that the preference of specific and general vegetable likers diverges across different types of vegetables. The reason is attributed to the complexity of sensory properties when vegetables are mixed with seasonings.” These results informed the cafeteria-based studies discussed below.

### Cafeteria-Based Attitudinal and Behavior Data

Our initial experiment in a cafeteria setting was designed to assess whether consumers would select seasoned or unseasoned vegetable side dishes when they were offered at no cost.^13^ An observational cross-sectional study was conducted over a 3-week period (November–December 2015), with 2 testing weeks and 1 washout week in between to ensure that each vegetable tested had the same entree pairing. Carrots, green beans, and broccoli were selected as test vegetables, based on their high consumption frequency by US adults.^16^ All customers purchasing a hot entree in the café, were offered a seasoned or unseasoned vegetable at no additional cost. The customers knew that they were participating in a study (they were later asked to fill out a questionnaire designed to assess: vegetable and seasoning status, vegetable liking, usual intake of the vegetable, usual intake of vegetables generally at lunch, likelihood of purchasing the vegetable, frequency of eating at the café, and demographics). One vegetable was offered per test day as both a seasoned and unseasoned choice, and vegetables were randomly assigned to a day of the week. Both the seasoned and unseasoned vegetables contained the same amount of olive oil and salt to enhance flavor. Industry experts in culinary sciences developed the seasoning blends that were unique for each of the vegetables tested in the café. Historical sales data from the study site was used to compare previous vegetable purchase patterns to selection in the current study.

Upon entry into the café, customers saw a display for the day’s food selections. As part of the selections, the seasoned and unseasoned vegetable dishes were displayed side by side with their respective ingredients under their name. Vegetables were labeled “seasoned” and “steamed” (for the unseasoned option) followed by the vegetable name. Although both seasoned and unseasoned vegetables were steamed, the term “unseasoned” was not used as a label to avoid an upward bias toward the seasoned vegetable option. Customers were not permitted to taste the vegetables prior to making their selections.

**Figure 3.**
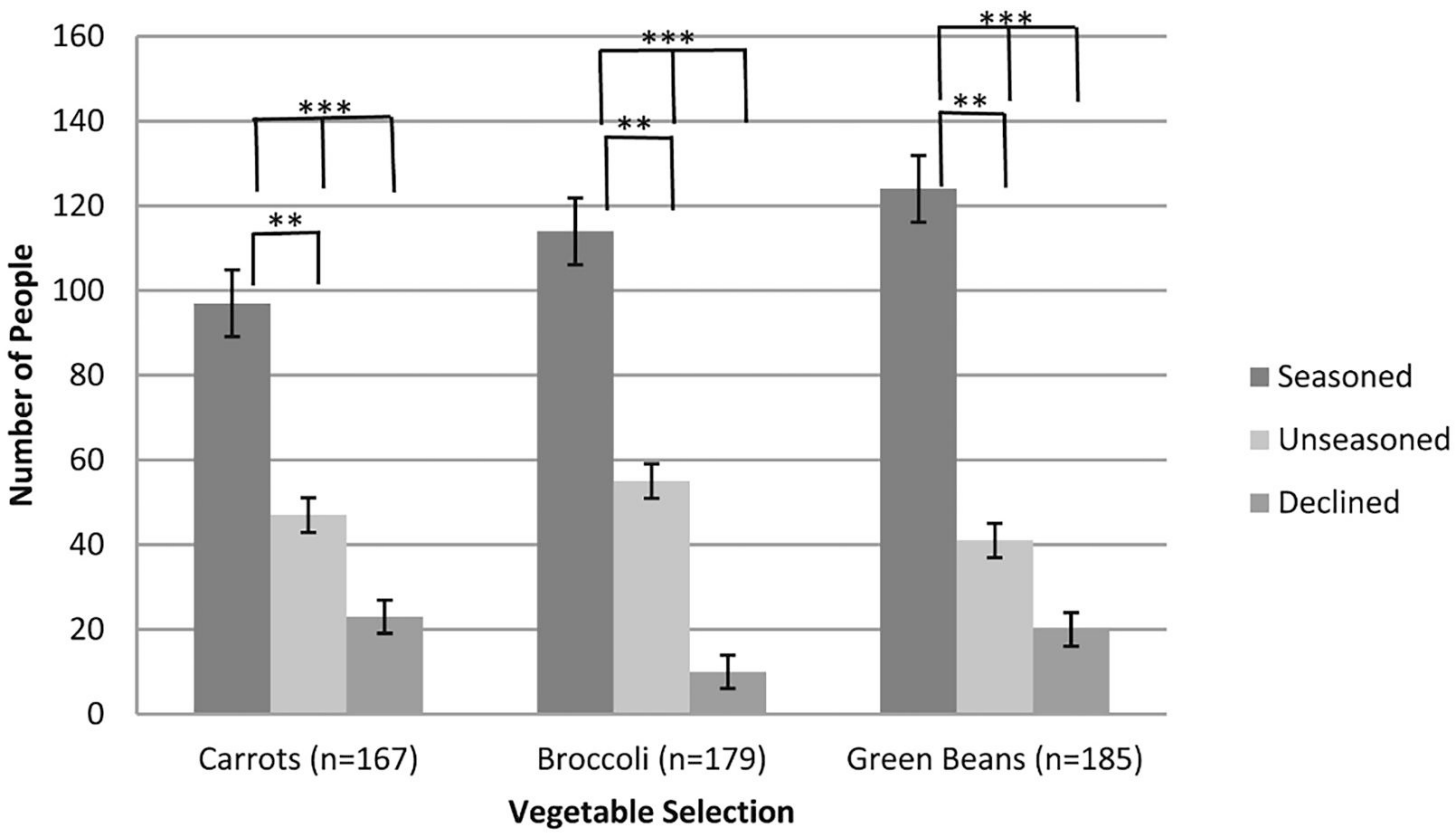
Selection of Vegetable Choice in a Cafeteria Setting. Source: Manero et al (2017)^13^

Most consumers selected one of the vegetable choices, with only 14% (*n* = 23), 6% (*n* = 10), and 11% (*n* = 20) of diners declining to take carrots, broccoli, and green beans, respectively. Figure 3 shows that seasoned carrots, broccoli, and green beans were selected significantly more often (carrots *P *< .001, broccoli *P *< .001, green beans *P *< .001) by consumers than the unseasoned versions (carrots: 58% [*n* = 97] selected seasoned, 28% [*n* = 47] selected unseasoned; broccoli: 63% [*n* = 114] selected seasoned, 31% [*n* = 55] selected unseasoned; green beans: 67% [*n* = 124] selected seasoned, 22% [*n* = 41] selected unseasoned).

To collect vegetable consumption data 4 oz (113.4 g) of each vegetable was portioned for each participant. Unconsumed vegetables were collected at the waste station and weighed in triplicate by student workers to determine the uneaten portion. The portion of vegetables not consumed was ≤20 g regardless of the preparation method (seasoned vs unseasoned). The vegetable and preparation method that produced the least waste was seasoned broccoli, with an estimated average of 5 g of waste per bowl. The vegetable and preparation method that yielded the most waste was seasoned carrots, averaging 20 g of waste returned per bowl.

It was concluded that, given a choice, consumers were more likely to select a seasoned vs unseasoned vegetable. This choice was based on preconceived ideas about what the descriptions (list of seasonings) meant to each individual, since sampling of the 2 options was not permitted. Nevertheless, despite the motivation for selection, it was concluded that offering seasoned vegetables may increase intake in those with poor vegetable intake in a café setting.

A second cafeteria-based study was conducted to determine whether the selection of seasoned vegetables compared with their unseasoned counterparts would be maintained when offered for purchase rather than free.^14^ Plate waste and intent to purchase larger portions were also assessed.

A cross-sectional study conducted in the same facility as our previous studies was used to evaluate consumer purchase decisions, liking, consumption, and larger serving purchase intention for vegetables. Each vegetable type was tested once a week for 8 weeks. Liking and likelihood of purchasing a larger serving size of steamed (steamed with oil and salt) and seasoned (steamed and mixed with oil, salt, and seasoning) vegetables were collected after lunch (11:30 AM–1:00  PM). Each participant completed a survey with questions regarding their experiences with the dish. Individual plates were weighed at the end of the day to assess vegetable consumption. Half cup (113.4 g) portions of steamed and seasoned vegetables were offered at the same price ($1.00). The $1 price point was used to be consistent with the actual price of other vegetable side dishes in the café.

Customers purchased significantly more seasoned green beans (*n* = 90 vs *n* = 44, *P *< .001), seasoned broccoli (*n* = 82 vs *n* = 54, *P *< .05), and seasoned cauliflower (*n* = 65 vs *n* = 22, *P *< .001) than steamed. Conversely, there was no significant difference in the purchase of steamed vs seasoned carrots (*n* = 38 and *n* = 30). As in our previous study,^13^ plate waste continued to be very low. The average waste per bowl for all vegetables was <6.5 g. Bowls of steamed green beans yielded the most waste, while steamed cauliflower yielded the least amount of waste. According to the ANOVA test, there was no significant difference in waste between steamed and seasoned across all vegetables, indicating that preparation methods did not affect consumer consumption of vegetable products. It is not known whether such differences would be observed with larger serving sizes.

The frequencies of the likelihood of buying a larger serving if either it cost the same or it cost more for each vegetable are shown in Table 2. There was an arbitrarily assigned incremental cost of $0.25 for the larger serving size. A χ^2^ test of independence was performed to examine the distribution of responses across all vegetable types. There was a statistically significant association between vegetable types and the likelihood of buying a larger serving. The relationships between these variables were significant (cost the same: *P* = .006; cost more: *P* = .018).

**Table 2.** Descriptive statistic of the likelihood of purchase a larger serving. Source: Luu et al (2020)^14^

	Cost was the same, *P *= .006^a^, *n* (%)	Cost was more, *P *= .018^a^, *n* (%)	
Green beans	Not likely	40 (28.0%)	53 (37.1%)
	Likely	103 (72.0%)	83 (62.3%)
Broccoli	Not likely	22 (15.8%)	32 (23.0%)
	Likely	117 (84.1%)	107 (77.0%)
Carrots	Not likely	10 (14.7%)	16 (23.5%)
	Likely	57 (83.8%)	51 (75.0%)
Cauliflower	Not likely	5 (5.7%)	13 (14.9%)
	Likely	77 (88%.5%)	68 (78.1%)

Not likely: Definitely would not, not likely. Likely: Somewhat likely, very likely, definitely would.

a Pearson χ^2^ for distribution of responses across vegetables. A total of 82.1% (*n* = 354) of participants were likely to purchase larger servings with no additional cost; 73.0% (*n* = 309) were likely to purchase larger serving with a $0.25 increase in price.

In conclusion, this café-based study found that all vegetables yielded negligible waste (<6.5 g/bowl); greater than half had 0 g waste. Participants were likely to purchase a larger size (cost the same: 82.1%; cost more: 73.0%). Seasoning was associated with more vegetable purchases for all vegetables. Participants liked the preparation method that they chose, eating most if not all. The results revealed that increased vegetable intake with larger servings may be possible.

A third observational study was conducted at the same facility to examine the effect of seasoning with spices and herbs on the consumption and liking of vegetables with larger serving sizes.^15^ Green beans, broccoli, cauliflower, and carrots were also used in this study. Similar to the previous studies, vegetables were offered as à la carte, but at a larger (1 cup) serving rather than the ½ cup (113.4 g) standard serving. In this study, the serving size was a weighed 8 oz (226.8 g) portion for all study vegetables during the 8-week period. The price was the same ($1.00) as for the previously served ½ cup portions and was based on the usual price of vegetable side dishes in the café. Vegetable bowls were collected at the waste station and weighed in triplicate by student workers to determine any uneaten portion. Participants were asked to respond to a survey modified from the previous study^14^ that included one item identifying the cooking method of the vegetable chosen, followed by an assessment of liking using a 9-point hedonic scale.

Similar to previous, seasoned vegetables were generally purchased more frequently than their unseasoned counterparts. When considering each vegetable separately, seasoned green beans, broccoli, and cauliflower were purchased significantly more often than steamed options (all *P <* .001). For carrots, there was no difference between the 2 preparation methods. Overall, when combining all vegetable types, the seasoned vegetables were selected significantly more than the steamed vegetables (*P <* .001). There were no significant differences in liking scores between steamed and seasoned vegetables within each vegetable type. However, liking responses were high overall, *M* = 7.3, indicating that consumers were highly satisfied with the vegetable dishes that they selected. A comparison of the wastes and intakes of different preparation methods and vegetable types showed no significant differences. Mean waste per bowl varied from 5.1 g (steamed cauliflower) to 30.9 g (seasoned carrots). Intakes were calculated by grams served minus waste. They varied from 221.7 g for steamed cauliflower to 195.9 g for seasoned carrots.

The significance of this study was that it examined the influence of larger serving size and seasoning on specific vegetables purchased as side dishes in a commercial cafeteria. It is promising that the combined methods resulted in a higher purchase of seasoned vegetables: green beans, broccoli, and cauliflower. Doubling the standard serving size successfully promoted vegetable intake by adult consumers, while retaining their acceptance of purchased vegetables at a high level.

Additional studies will be necessary to fully understand the ability of spices and herbs to affect vegetable consumption. The discrepancy between the controlled sensory panel data, which generally showed seasoned vegetables to be preferred compared with their unseasoned counterparts, and the general lack of such preferences in the cafeteria studies deserves further exploration. It may be that consumers had a propensity to assign a high acceptance rating to the particular form of vegetable they selected (thereby reinforcing their own decision) contributed to this outcome. In addition, the fact that there was very little plate waste regardless of the serving size of vegetable provided suggests studies with even larger serving sizes would be necessary to fully understand the impact on consumption. Nevertheless, from a pragmatic standpoint, the existing data showed that combining seasoning and doubled serving size effectively increased selected vegetable intakes, and could be a viable strategy for increasing vegetable consumption in a café setting.

## Data Availability

The data underlying this article are available in the article and in its online supplementary material.
